# How and why buy-in for health in all policies was facilitated in Ecuador: a realist case study of *Plan Nacional para el Buen Vivir*

**DOI:** 10.1186/s12939-022-01703-7

**Published:** 2022-08-15

**Authors:** Deb Finn Mahabir, Ketan Shankardass, Alix Freiler, Patricia O’Campo, Ben Brisbois, Carles Muntaner

**Affiliations:** 1 MAP Centre for Urban Health Solutions, 30 Bond Street, Toronto, ON M5B 1W8 Canada; 2 Department of Health Sciences, Wilfrid Laurier University, 75 University Avenue West, Waterloo, ON N2L 3C5 Canada; 3 School of Health Sciences, University of Northern British Columbia, 3333 University Way, Prince George, British Columbia V2N 4Z9 Canada; 4 Faculty of Nursing, University of Toronto, 155 College Street, Toronto, ON M5T 1P8 Canada; 5 Dalla Lana School of Public Health, University of Toronto, Ontario, M5T 1P8 Canada

**Keywords:** Ecuador, Health inequities, Extractivism, Health in all policies, Healthy public policy, Implementation, Realist methods, Social inequities, Systems theory

## Abstract

**Background:**

In 2008, Ecuador introduced *Plan Nacional para el Buen Vivir (PNBV;* National Plan for Good Living), which was widely recognized as a promising example of Health in All Policies (HiAP) due to the integration of policy sectors on health and health equity objectives. PBNV was implemented through three successive plans (2009–2013, 2013–2017, 2017–2021). In a time of widening global health inequities, there is growing interest in understanding how politics and governance shape HiAP implementation. The objective of this study was to test specific hypotheses about how, why, to what extent, and under what circumstances HiAP was implemented in Ecuador.

**Methods:**

An explanatory case study approach (HiAP Analysis using Realist Methods on International Case Studies—HARMONICS) was used to understand the processes that hindered or facilitated HiAP implementation. Realist methods and systems theory were employed to test hypotheses through analysis of empirical and grey literature, and 19 key informant interviews. This case study focused on processes related to buy-in for a HiAP approach by diverse policy sectors, particularly in relation to the strong mandate and transformative governance approach that were introduced by then-President Rafael Correa’s administration to support PNBV.

**Results:**

The mandate and governance approach of the HiAP approach achieved buy-in for implementation across diverse sectors. Support for the hypotheses was found through direct evidence about buy-in for HiAP implementation by policy sectors; and indirect evidence about allocation of governmental resources for HiAP implementation. Key mechanisms identified included: influence of political elites; challenges in dealing with political opposition and ‘siloed’ ways of thinking; and the role of strategies and resources in motivating buy-in.

**Conclusion:**

In Ecuador, political elites were a catalyst for mechanisms that impacted buy-in and government funding for HiAP implementation. They raised awareness among policy sectors initially opposed to PNBV about the rationale for changing governance practices, and they provided financial resources to support efforts related to PNBV. Specific mechanisms help explain these phenomena further. Future studies should examine ways that PNBV may have been an impediment to health equity for some marginalized groups while strengthening HiAP implementation.

## Introduction

As a governance concept, Health in All Policies (HiAP) is a process of making “formal and sustained use of structures, mechanisms, and actions that are managed mainly *outside* of the health sector to improve population health and reduce health inequities across social groups” ([1], p. 8, emphasis added). At a time of widening health inequities worldwide [2, 3], Ecuador’s National Development Plan for the implementation of social policies and related institutional changes – the National Plan For Good Living or *Plan Nacional para el Buen Vivir* (PNBV) – was widely recognized as a promising approach to HiAP because of the integration of policy sectors through a system of coordinating ministries [1, 4]. The World Health Organization asserts that a HiAP approach is central to improving health equity [4]. More recently, there has been growing interest in understanding how politics and governance affect HiAP implementation [5–7].

Implementation of HiAP usually represents an innovation in governance, which can make it both technically and politically challenging for governments to utilize. Specifically, HiAP requires individuals across diverse government sectors to understand that major determinants of health and health equity are under their influence; and then learning how to manage these determinants in the policymaking process alongside other governmental and non-governmental actors [8]. Implementation of HiAP is thus a “deeply *political* process that involves conflicts over power, resources, and ideological assumptions about the importance of the state and the market in achieving social objectives” ([5], p. 745, emphasis added).

To examine the implementation of HiAP, we used a previously developed realist method for studying the implementation of HiAP [9], and employed a systems theory framework (discussed further in the methods section). Briefly, to better understand how and why – and to what extent – Ecuador’s approach to HiAP led to changes supportive of health equity, we used a realist explanatory case study of the implementation of PNBV (recognized as an example of HiAP by the World Health Organization [4]), a new constitution that introduced a novel governance structure (i.e., coordinating ministries). We were particularly interested in identifying political mechanisms (hereafter, mechanisms) related to the adoption of which reflects a strong mandate for HiAP. Our methodological approach includes a framework of HiAP sustainable implementation outcomes of key interest, including how and why political mechanisms supported or hindered acceptability and feasibility of Ecuador’s HiAP implementation across diverse sectors [9]. We also used a systems theory framework of HiAP implementation (see Shankardass et al. [10]) to understand inter-relationships between mechanisms. Finally, to test hypotheses about specific mechanisms and learn more about the implementation of HiAP in Ecuador, we used triangulation of evidence about specific mechanisms. To better understand the context in which these mechanisms are embedded, we turn next to the political tradition or ideology of the Correa government.

### The emergence of HiAP in Ecuador: a popular reform

Since the global economic oil crisis of 1980, there has been an erosion of state social protection and redistributive policies in many Latin American countries [11, 12]. Such policies have often been considered obstacles to economic growth and in conflict with neoliberal macroeconomic policies imposed on the region [13]. In 2005, the Ecuadorian presidential election campaign took place in the context of high levels of poverty; in 2004, 52% of the total population was living in poverty with another 14% in extreme poverty [14]. To address growing dissatisfaction with austerity measures and multinational companies’ outsized roles, presidential candidate Correa ran on a platform proposing an end to the "long, sad night of neoliberalism” ([15], p. 275). Correa came to lead a political movement that centred on political sovereignty, regional integration, and poverty relief, fuelled by greater national control over and extraction of natural resources [16].

Originally, *buen vivir* (BV) is an Indigenous Ecuadorian Amazon concept stemming from the notion of *sumak kawsay* meaning “limpid and harmonious life” ([17]. p. 11). It views nature and social environments as inseparable and fundamental to well-being and intergenerational sustainability and was part of the Amazaga Plan of the Organization of the Indigenous Peoples of Pastaza [17]. After winning the 2006 election, Correa oversaw the development of a new Ecuadorian Constitution drawing on the concept of BV as an alternative to previous neoliberal policies. This new constitution saw “equity as a governing principle” ([18], p. 54), and implementation was started through the corresponding comprehensive National Development Plan 2009–2013. Consisting of 12 strategies, 12 national objectives and 92 policies, this plan included the objective “to improve the quality of life of the population” and articulated a strong focus on poverty reduction, health, and reforms to public institutions ([18], p. 76).

Weisbrot et al. (2017) [19] report on social gains during Correa’s time in office, from 2006–2016. These include 38% and 47% declines in poverty and extreme poverty, respectively, including through direct cash transfer. There were also reductions in income inequality, with the Gini coefficient shrinking from 0.55 to 0.47 over this period. The government also increased social spending (including education, health, and urban development and housing) over this period, from 4.3 percent (2006) to 8.6 percent (2016), as a percentage of gross domestic product.

On the other hand, progress on some important social indicators was questionable during Correa’s administration. Infant mortality decreased during his first term in office but the decrease slowed after 2012 and the period after 2015 actually saw increases in neonatal, postnatal, and under-5 mortality [20]. Women’s and health justice groups have highlighted a lack of strong engagement by Correa’s administration with social organizations representing sexual and reproductive rights, despite Ecuador’s continuing high rates of teen pregnancy [21]. While health spending increased substantially during Correa’s administration, critics maintain that this was inappropriately focused on treatment instead of prevention and was accompanied by massive privatization. Indeed, some argue that although social spending, including health spending increased during Correa’s administration, neoliberalism was not fundamentally addressed, weakening the impact on health inequity of PNBV [22].

In this analysis, we do not focus on the extent to which PNBV impacted on health equity in Ecuador. Instead, we focus on understanding how Correa’s approach to leadership helped to transform governance in Ecuador to reflect a HiAP approach. The political tradition or ideology of a government impacts the policies that are implemented and thus partly determines levels of economic and health inequities [23]. In the case of Ecuador, the Correa government aimed to address inequities using the PNBV strategy, supported by institutional changes to integrate the work of policy sectors (i.e., ministries) through a newly introduced system of coordinating ministries [1, 4]. Hence, Ecuador presents an interesting case study of whether strong political commitments for health equity as part of a broader development strategy lead to the successful implementation of a HiAP policy orientation.

Given that the ideological orientations of political parties ruling with a majority can have a significant effect on political agendas and thus support or hinder HiAP implementation [6], the objective of this paper is to test our hypothesis about the role of the strong mandate for PNBV to increase buy-in for HiAP implementation by identifying mechanisms related to the implementation of PNBV in Ecuador. Specifically, we hypothesized that the strong mandate for PNBV adopted by Rafael Correa’s popular government during the start of his administration would lead to increased buy-in for implementation across diverse sectors.

## Methods

### Realist explanatory case study methodology

A realist explanatory case study methodology (adapted from Yin [24]) was used as a reproducible systematic theory-informed approach to identify and analyse mechanisms that hinder or support HiAP implementation. Previously, case studies have been used primarily for observational or descriptive research questions. In this study, we used a realist explanatory (causal) case study approach as described by Shankardass et al. [9]. Interviews and the literature were analysed for context-mechanism-outcome (CMO) pattern configurations as developed by Pawson and Tilley [25].

The methodology consisted of several steps. First, an initial review of the literature: key concepts from political, policy, and public health sciences were examined to understand the general role of politics in influencing HiAP implementation [6]. Second, findings from the review were used to develop hypotheses about HiAP implementation mechanisms. Third, a systematic review of the grey and scholarly literature was then conducted on HiAP implementation in Ecuador; a total of 16 articles in English and 3 articles in Spanish were reviewed (for the search strategy, see Shankardass et al. [1]). Fourth, semi-structured interviews with key informants were conducted. Last, data from steps 3 and 4 were analyzed for CMOs following an approach described in Shankardass et al. [26]. Data extraction of CMOs were conducted by the research team working in pairs. Each review team presented their extraction results with the entire research team, including discrepancies and justifications for inclusion or exclusion in order to support transparency, consistency, and feedback. This realist approach to identifying CMOs is supported in the literature [27].

### Initial theory: conceptual framework for the sustainable implementation of HiAP in Ecuador

**Fig. 1:**
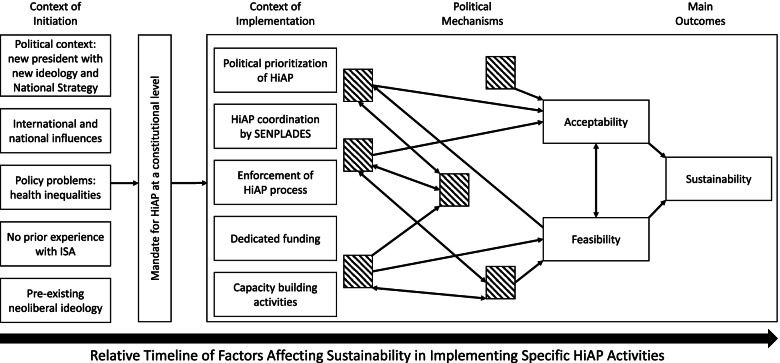
Conceptual framework for sustainable implementation of HiAP in Ecuador

Given the objective of articulating mechanisms, realist scientific approaches are strengthened by the development of an initial theory. Our realist explanatory case study methodology also used initial theory as a basis for developing hypotheses to test in the analysis. To develop an initial theory, we adapted the conceptual framework initially developed by Shankardass et al. [9] using the literature specific to Ecuador during the implementation of PNBV and from previous HiAP case studies (e.g., Molnar et al. [27]) to conceptualize contextual factors about the initiation and implementation of PNBV that influenced the sustainability of HiAP implementation in Ecuador (Fig. 1). Data collected during the case study were then used to identify mechanisms of HiAP implementation in Ecuador. Finally, using a systems theory framework [10], we then analyzed unique government structures relevant to implementation mechanisms in Ecuador.

The theory-informed framework developed by Shankardass et al. [9] operationalizes an understanding of how the context of initiating and then implementing HiAP affects mechanisms that can cause multisectoral acceptability and feasibility of HiAP implementation, and thus, sustainable implementation. The adapted framework presented below guides an understanding of the context, mechanisms, and outcomes of the political process at the start of Ecuador’s HiAP mandate (i.e., the adoption of PNBV) that positively and negatively affected HiAP implementation. We also examined how and why new coordinating ministries impacted HiAP implementation. Below, to situate our results, we define and describe contextual factors in general and then as they relate to Ecuador within the period of 2009 to 2013.

### Context of initiation: political elites

In the context of initiation of HiAP in general, political elites are defined as individuals who have influence over the design, implementation, orientation, and evaluation of HiAP. Political elites at national and local levels or within different sectors may have different ideologies and agendas, which may result in ideological and jurisdictional conflict across sectors being engaged in HiAP implementation [28]. The political agenda is understood as a set of cultural, economic, and political patterns of decision making within a political system, which is influenced by ideology (ideas influenced by values) and used by political elites to assert the role of the markets, states, and individuals in achieving health equity [6].

### Context of implementation: Acceptability and feasibility for sustainability

In order for HiAP interventions to be implemented sustainably, there has to be on-going buy-in from partners across government sectors. Achieving buy-in from a range of health and non-heath sectors is a function of inter-related actions and activities that promote acceptability (i.e., willingness of sectors to collaborate on health and equity objectives) and feasibility (i.e., capacity of sectors to collaborate), and contribute to the continuation of HiAP implementation. Jurisdiction refers to all levels of government and considers how authority and political responsibility for policy issues are formally distributed [6, 29].

### Context of initiation and implementation specific to Ecuador

In Ecuador, support for the HiAP mandate stemmed from the president, an elite politician, through a series of national strategies and priorities identified in PNBV. Policy coordination and integration was supported by the National Secretariat for Planning and Development or *Secretaría Nacional de Planificación y Desarrollo* (SENPLADES) and funding for intersectoral activities was provided through an integrated budget. Integrated governance was also supported through the National Planning Council, an intersectoral, professional body that functioned as the technical secretariat, providing expertise to all levels of government. The Coordinating Ministry of Social Development had a supervisory role over the Ministers of Health, Labour, Inclusion, Migration, and Housing, and provided oversight regarding participation of non-health sectors at all level of government in PNBV [4, 18].

### Systems theory framework

We used a systems theory framework of HiAP implementation to support our understanding of mechanisms as emergent properties arising due to unique interactions and relations between subsystems and specific components [30]. The use of systems theory is recognized as important to understanding the complexity of health inequity [31].

Our systems theory framework [10] was empirically informed and has been used by our study to support hypothesis development and testing, as well as data analysis. This framework is comprised of three interdependent government subsystems: executive (heads of state/government and other political elites); intersectoral (policymakers and experts working with governance structures related to HiAP that facilitate horizontal policy coordination); and intrasectoral (policymakers within policy sectors). There are also extra-governmental influences (outside of the government system) that interact with these subsystems.

### Data sources

A total of 19 key informants (politicians and civil servants representing different sectors, academics, and activists/advocates) living in Ecuador were recruited. In keeping with a realist approach, our hypotheses represent our initial theory about HiAP implementation. Developing an initial theory or set of initial hypotheses is a fundamental first step in a realist method approach [27]. This initial theory guided the development of our semi-structured interview guide about barriers and facilitators to HiAP implementation. Interviews were conducted in Spanish between December 2014 and May 2015 and then translated to English.

### Data analysis

To support study rigour, we triangulated data from multiple sources characterizing the early phase of HiAP implementation (2009–2013) including scholarly and grey literature, and key informants from different sectors. CMOs were categorized as ‘thick’ or ‘thin’ support for the hypothesis based on the quality of the description provided by key informants or in documents (literature). Thick CMOs provide a rich or detailed description of the context and mechanism(s) that led to a specific outcome during HiAP implementation, whereas thin CMOs lack critical details about one or more components of a CMO. Thin CMOs were deprioritized for reporting since they were incomplete and thus, according to scientific realist methods, could not be confirmed as real. Given that our main interest is to support an understanding of mechanisms in context that facilitate or hinder HiAP implementation outcomes, this paper focuses on thick CMOs.

We also assessed the degree of support for our hypotheses about HiAP implementation via multiple types of triangulation. We tabulated the number of thick CMOs for each hypothesis. Also, the degree of support for the hypothesis was considered strong when there was thick evidence from three or more sources of data (e.g., literature versus key informants, as well as key informants representing different sectors or perspectives); adequate thick evidence was from two sources of data; limited thick evidence was from only one source of data.

To understand what mechanism(s) triggered a specific outcome that supported or hindered the acceptability and feasibility of HiAP implementation, once an outcome was uncovered from an interview or literature data, specific mechanisms and contextual factors were identified (if provided) to describe the CMO configurations. We organized our results according to HiAP implementation outcomes. There were five broad outcome categories: breadth of sectoral participation, governmental funding of resources, introduction of government structures, content of HiAP interventions, and feasibility of implementation.

## Results

Overall, we found strong support for our hypothesis that the mandate for HiAP adopted by Correa’s government resulted in increased buy-in for HiAP implementation across diverse sectors. In particular, we learned that commitment and political leadership by Correa facilitated buy-in, in part because this leadership led to the allocation of government resources that enriched HiAP implementation. Findings are organized by support for these two inter-related outcomes: buy-in for HiAP implementation and allocation of governmental resources for HiAP implementation. Buy-in is understood as a function of inter-related actions and activities that promoted acceptability and feasibility (i.e., policy coordination and integration support by the National Secretariat for Planning and Development) and contributed to HiAP implementation in Ecuador. Allocation of government resources is related to buy-in and refers to how resources (e.g. funding for intersectoral activities through an integrated budget in Ecuador) were distributed among competing demands based on the new HiAP mandate: PNBV. These outcomes were part of our conceptual framework for sustainable HiAP implementation, which was used in the development of the initial theory specific to Ecuador (as discussed in the methods section).

### First outcome: buy-in for HiAP implementation

#### Mechanisms that facilitated buy-in

We identified three mechanisms that facilitated buy-in. In the first mechanism, political elites, including the president and other ministers, were influential in HiAP implementation because of their formal authority (a form of power) to facilitate changes in governance (i.e., the introduction of coordinating ministries) that resulted in a new decision-making hierarchy. There was strong evidence from four key informants that this new governance structure was used to support PNBV objectives related to social sectors, which increased buy-in for HiAP implementation across some sectors.

For example, one key informant explained that “if there was different thinking between the Finance Minister and the Health Minister related to funding allocation, we would go to the president for a decision…the finance ministry did not have the same power that finance ministries have in other countries. We had the possibility of reaching the president directly to iron out differences with the finance ministry”.

A non-health sector key informant explained how clear support from the president (Correa) led to buy-in for implementation; since the president had ultimate decision-making authority over the implementation of the policy agenda, he also had the power to compel sectoral participation in PNBV specifically. This key informant clarified that institutions and ministries “generate [many] proposals, but they are not implemented without the president’s approval. When it comes to difficult processes of change, [decisions] are always centred on him”.

A second mechanism through which political elites facilitated buy-in for HiAP implementation was their leadership style (such as engaging partners and seeking participation) and influence. This mechanism also had strong support in our data, including four key informants who explained how political elites used this mechanism at national and local levels to facilitate policy coordination and buy-in for implementation.

When speaking about the influence of a charismatic personality and the leadership of a political elite, one non-health sector key informant commented that “there are leaders at all levels. There are some cases of some ministers that exert an unquestionable leadership. Minister (x) is a heavy as we say, so he does achieve a lot of coordinated work; when he wants something almost no one refuses. When the ministry needs to coordinate some work with others, the minister´s request is very much considered”.

We also learned how local leaders achieved buy-in for HiAP implementation through this mechanism: “at the local level there are many that have human quality and great leadership; these are the leaders that go to meetings and exert leadership, they are democratic, they seek participation. So, this type of leaders have [sic] a pull, they bring institutions together to work in coordination”.

Additionally, it was claimed that name recognition was one of the reasons why some leaders exert strong positive leadership: “Because of their historic connotation; they are people that have done good to the country all their lives, so people consider them; they have them in high regard…unquestionable leadership”.

Another leadership characteristic that was influential in supporting buy-in was described as a leader's ideological strength. This key informant noted that these leaders “engage people; they are ideologically strong, and they have charisma”. One non-health sector key informant explained how the president’s ideology was part of his leadership style that influenced buy-in with the cabinet as it supported their ability “to believe in a different country, with different opportunities and whose development is based on a solid social basis”. The president “has a very strong conviction that to have a developed country it is necessary to invest in policy and social programs. He has spoken about this. So, I think he has been the main leader in maintaining a sustained development in the social sector. I also think all the cabinet [members] have a total conviction that as long as we can expand the capacities and our population and generate more opportunities, we will be able to materialize that dream that we had when we put together PNBV and the constitution”.

The third mechanism that facilitated buy-in was a motivation for HiAP, which supported implementation; sectors’ awareness of the rationale for HiAP led to an understanding of their contributions and how they could coordinate their policies to improve public health outcomes. Prior to the constitutional change and implementation of PNBV, sectors worked independently towards their objectives. There is strong evidence that once sectors understood how they could contribute to the improvement of public health, they were motivated to support the development and implementation of broader social policies. Three key informants explained how the coordination of policies occurred.

At the national level, a non-health sector key informant identified that once individuals understood their specific work contributions towards public health outcomes and, in particular, how they could coordinate their interests with those outcomes, sectors were motivated to participate, which then led to buy-in. This key informant provided some background: “Health was always managed by the health ministry, same with education, same with economic and social exclusion. The main challenge has been coordinating and articulating work around certain issues”. This key informant then explained the importance of acknowledging the historical context of policy implementation as a way of moving forward towards the coordination of current policies: “We respect each sector’s autonomy. They rule over their own jurisdiction. But there are issues that require the intervention of different sectors. Once you understand the issue this way, it’s easier to work in coordination. You don’t take the protagonist role away from sectors. Since the result will be measured, the sectors end up following because they know that if they work alone they will not achieve the results”.

At the local level of HiAP implementation, a participatory process was used to raise awareness of HiAP guidelines from the national to the local levels. To create ownership and policy coordination, it was important to engage local actors. One non-health key informant asserted that “the only way to ground policy is if you have engagement and participation from local actors”. This key informant explained that from experience, “it is different if you go to the provinces and meet with people that know their services and take ownership of policies, and to (then) build services that are directly linked to their expectation. It’s not always easy because we are interested in changing these structures very fast, so it’s difficult; however, it’s about not losing contact with those that sustain the process”.

### Mechanisms that hindered buy-in

Two mechanisms were identified as hindering buy-in. The first mechanism involved initial opposition to HiAP implementation by policy agents from some sectors due to conflicting interests, which slowed down the agenda setting process for policy implementation. We found strong evidence from five key informants for this mechanism of jurisdictional conflict during the initial implementation of PNBV at the national level. In 2008, during the implementation of PNBV, when coordinating ministries were introduced into the government (integrated governance) some ministries were required to take on new responsibilities. Evidence from three key informants demonstrates that some sectors opposed changes that impacted their relationship to corporate interests and thus sectors were protective of their control over their jurisdiction.

In one CMO, one such clash occurred between the pharmaceutical industry and a new regulatory agency. As part of an effort to make medication accessible to the public, the government introduced a regulatory agency with jurisdictional control to lower medications costs. This caused concern in the pharmaceutical industry about loss of control over medication pricing and hence loss of profits. As explained by a health sector key informant, there were “certain privileges that some groups enjoyed in this country, in the private sector as well as the provision of health services. They had a really interesting growing market, and then the moment this policy of HiAP came in, of universal access to health in any/all circumstances, some very major economic interests were affected”. In this example, a possible decrease in profits for the pharmaceutical company hindered buy-in.

Another health sector key informant described how the shift to integrated governance led to proposed changes in food labelling design that were met with opposition by other sectors due to an increase in costs. As this key informant explained:

The discussions that happened with the Ministry of Production were about interests. At a given moment what got approved was for this labelling to be in the front part of the product, and then they said it had to be on the back. The reason is that there are economic interests there. I think intersectoral work is limited by those elements. In the case of food labelling, this causes debate and a political overtone to the discussion the whole time.

One key informant described that “before 2007, Ecuador was like nobody’s land. Everything was distributed amongst private interests. Perhaps for profit, perhaps for non-profit. But there was no government structure and even less a social structure”. In 2008, during the implementation of PNBV, integrated governance through coordinating ministries required that some ministries take on new responsibilities. Other key informants explained that as a result of changes made in support of integrated governance, some workers within sectors feared a loss of job autonomy. One non-health sector key informant explained that there was “fear of losing control over areas of work; certain functions were duplicated, and people wanted to defend what they thought was theirs”. As noted by another non-health sector key informant: “People were afraid of potentially losing their job if things were done differently”. A key informant added that “this changed little by little, until we got to the culture where all sectors make an effort so that no topic is left behind, and things are done in an integral way while respecting everyone’s area of work”.

The second mechanism that hindered buy-in was an initial opposition to HiAP due to a lack of awareness; this meant that participation required an awareness of the need for HiAP to support implementation. Strong evidence demonstrates that an initial lack of workforce awareness of the need for HiAP among government sectors impacted the ability to engage in implementation, which hindered buy-in. Five key informants and one document supported this finding. As one health-sector key informant explained, “Sometimes changing the way they [people in the sectors] work causes resistance until people become aware of the fact that the new ways of working are what is needed to make changes. Things gradually fell into place because deep inside people knew it was necessary to make some radical changes to both the structures and the processes that happened inside institutions”.

A non-health sector key informant noted that at the level of the provinces, there was initially a failure to develop adequate awareness of information systems, protocols and procedures, and tools at the sectoral level (e.g., lack of training for front-line service providers) for the operationalization of intersectoral action (ISA). ISA is understood as a relationship between parts of the health sector with parts of another sector that work together on a specific issue (e.g., tobacco control) or on a broader concern (e.g., overall quality of life) [27]. As the key informant explained:

So, for example, sometimes a simple list, or a system, or information system or simply not knowing the protocols or the intervention procedures. It is as if all this becomes formal at the national level, but the provinces either don’t have the information or they refuse to use it. It’s about giving them that awareness. They don’t receive the proper training. Many times, they think that if they send the front line a text, they will read it and they will learn, but it is a process. And if you don’t accompany them in it and if you don’t train them to implement it properly up to the point where you see that it happens, it simply doesn’t happen.

### Second outcome: allocation of governmental resources for HiAP implementation

For this second outcome, which is related to facilitating buy-in, one mechanism was identified: With dedicated funding for HiAP supported implementation, the political agenda changed resource allocation prioritization. Strong evidence indicated that the national government used its influence to support the objectives of social sectors through an increase in social investment. In Ecuador, health was named as a right in the constitution. Six key informants and one document explained how the political priorities of the new government served to preferentially increase funding for health and education.

At the national level, restructuring created a new national norm. Specific HiAP funding initiatives at the national level focused on priority investment projects such as health and education. Conversely, other sectors were less well funded since they did not have the same prioritization. A non-health sector key informant explained why PNBV strategies allowed for a focus on poverty reduction. This key informant explained that “they focused on high priority areas that were quite weak. Health and education in Ecuador were worrisome, so they were prioritized”.

Another non-health sector informant detailed that “we adjust the budgets that are assigned each year to the sectors. The goals are determined by factors such as increasing access to services, improving the quality of health care service, reducing the gaps in different provinces”. This key informant explained how an agreement on resource allocation was achieved: “Based on that, we agree on the necessary activities and then we get together with the president. He has a say in the priorities that have been established by each sector. We respond to issues not as individual sectors but as social fronts that respond to national objectives”.

As explained by another health sector key informant “when things don’t work, a lack of resources is not an excuse. There was a clear priority [an order] from the government that says that things that are planned for high priority activities will not lack resources”.

## Discussion

Using our systems theory framework [10], (as presented in the methods section), we now explore how and why the practices of political elites and policy-makers from specific governance structures and policy sectors facilitated or hindered Ecuadorian implementation of HiAP; for clarity, system components from the systems theory framework will be *italicized*. Overall, our analysis indicates that the executive sub-system in Ecuador was a catalyst for mechanisms that impacted buy-in and government funding for HiAP implementation. Changes made to government institutions to support the implementation of the new political agenda created a new working dynamic that facilitated implementation outcomes in key ways. Within the executive sub-system, *political elites* appear to have been particularly influential regarding the political ideology that guided changes in the *policy agenda*, *HiAP mandate* (PNBV), and related *HiAP financial arrangements* or funding initiatives. In particular, political elites raised awareness in sectors initially opposed to HiAP, which motivated participation in intersectoral collaboration. Political elites also supported dedicated governmental funding for the political agenda, which facilitated buy-in through various mechanisms. These mechanisms specific to the role of political elites, initial opposition, motivation, and dedicated funding on buy-in, are discussed below.

### Driving role of political elites

*Political elites* used their formal authority to change the structure of ministries in a way that compelled sectoral participation and thus buy-in for implementation. Additionally, elites sometimes used their formal authority to allocate resources in keeping with the dominant *political ideology* to support the objectives of social sectors engaged in PNBV. We also found evidence that political elites at national and local levels influenced the implementation of HiAP by using their leadership to engage partners to buy-in. Our initial theory about political elites however, did not identify nor test specific leadership styles and its impact on HiAP implementation. Given our key finding that political elites play an influential role in HiAP implementation, an examination into different leadership styles of political elites warrants further investigation. Such an examination would support a better understanding of the leadership characteristics that support or hinder HiAP implementation.

### Role of initial opposition to HiAP in sectors

Diverse sectors viewed the new objectives and processes as being in conflict with their previous *sectoral objectives* and *sectoral power*. We found evidence that a perceived loss of autonomy implied by integrated governance was viewed as a potential challenge to the traditional jurisdictional control between sectors, hindering buy-in. To overcome this opposition, HiAP management strove to build *workforce capacity for intersectoral action* by instructing sectors about the new processes and training required for implementation and raising awareness of the need to engage with different sectors. Raising awareness and building capacity reduced jurisdictional conflicts and facilitated buy-in.

### Role of creating motivation within sectors for HiAP

With a long history of *sectoral power*, getting sectors to break from long-standing siloed ways of thinking created tensions and initially negatively influenced buy-in. However, continued efforts to develop *workforce HiAP awareness* about the importance of working intersectorally to address health equity eventually promoted buy-in. At both national and local levels, professionals in diverse sectors required a better understanding of how their sector could coordinate their policies to improve health outcomes. Once this understanding was established, some sectors were motivated to improve public health outcomes, facilitating buy-in.

### Role of dedicated funding for the political agenda

A systems theory analysis makes evident how a political elite – Correa – motivated buy-in through dedicated funding for HiAP initiatives. Correa had the ultimate decision-making authority over the new *policy agenda*, and as a result, changed the resource allocation of funds. Correa identified health and education as important, which gave it strategic importance and made it a priority for *HiAP financial arrangements.* Indeed, when the National Development Plan was first initiated, social investment increased 2.5 times [4].

Overall, our findings in Ecuador are consistent with the available emerging HiAP literature. In Sweden, Molnar et al. [27] found that raising awareness was effective to motivate sectors for HiAP implementation only when specific initiatives were funded. Kokkinen et al. [32] observed that, in Finland, without formal funding allocation for HiAP, the capacity of national authorities to support HiAP activities diminished, resulting in poor outcomes within different municipalities. These recent findings highlight the importance of dedicated funding towards supporting and sustaining HiAP implementation.

Although our study focused on understanding how the strong mandate and transformative governance of PNBV affected HiAP implementation in Ecuador, recent scholarly and grey literature that was largely not captured by our methods highlights additional mechanisms that compromise the health equity potential of PNBV. The undeniable positive short-term impacts of PNBV included a reduction of poverty in Ecuador from 46 to 30% and indigence (extreme poverty) from 19 to 9% between 2007 and 2014, but while inequality also decreased from a Gini coefficient 0.547 to 0.476, the distribution of land and productive assets in the country remained largely unchanged [33]. Recent studies have also demonstrated continued inequities in Ecuador based on ethnicity, income, occupational class, education, and region [34–36]. In addition, numerous voices have critiqued the ways in which the social programs implemented by PNBV followed an ‘extractivist’ development model based primarily on increased resource extraction to fund poverty reduction and social spending [37]. While the Correa government did introduce some progressive taxation measures redistributing wealth, these were not nearly enough to fund the social programs mandated by PNBV, which instead were financed by a combination of historically high commodity (e.g., oil) prices and borrowing, especially and increasingly from China – backed by commitments of future mining and petroleum exports to Chinese creditors, thereby ‘locking in’ the extractivist development model [38]. When commodity prices dropped after approximately 2014, the Correa administration introduced austerity measures, while rising debt levels were among the factors motivating his successor President Lenin Moreno, to make further cuts to the social programs in PNBV. National protests in response to a package of such austerity measures required under an International Monetary Fund (IMF) loan agreement brought the country’s economy to a standstill in late 2019, while cuts to the health sector made by Moreno in connection with the IMF package have been identified as a major reason for Ecuador’s disastrous experience of the COVID-19 pandemic [39].

Additional caveats regarding the overall health equity benefits of PNBV come from sustained Indigenous and civil society opposition to the imposition of resource extraction projects held necessary to fund social spending [37]. The Correa government became notorious for criminalizing and demeaning such voices, which had previously helped bring it to power [38]. One qualitative study [40] identified a group of stakeholders outside of the public sector and not involved in the implementation of PNBV who were more critical of the implementation of PNBV. For example, one stakeholder was critical of Correa’s turn towards extractivism in 2012, which is echoed in other articles that highlight his government's aggressive promotion of mining, oil and gas extraction, and agroindustry [16, 37]. In another interview, a government stakeholder argued that extractivism in the short term was necessary to finance a reduction in poverty [40], echoing a common narrative in Correa’s public service (and public speeches) that helped to dismiss resistance to resource extraction as ‘infantile’ or insufficiently informed by science [38].

Such dismissals (and frequent violent repressions) of Indigenous protest, and documented negative impacts of resource extraction projects imposed in Indigenous territories, also problematize the appropriation of the Indigenous notion of BV by the Correa government [41]. Importantly, such perspectives were largely absent from the sources of data we analyzed, with the exception of one author’s account – which only just reached our criteria for ‘thin’ mechanisms – of how education policy reform under PNBV reduced the power and budget of the National Office of Intercultural Bilingual Education to implement a ‘plurinational’ and ‘intercultural’ bilingual system of education [42]. This reform by the Ministry of Education resulted in the replacement of Indigenous teachers by *mestizo* (of mixed European and Indigenous ancestry) teachers; and the removal of Indigenous organizations from the management of bilingual schools. This author [42] posits a mechanism in which Indigenous peoples “are reduced to a folkloric representation, suitable to the consolidation of the capitalist model based on the mining and petroleum extractivism of the transnationals” ([42], p. 82).

Such observations, coupled with the numerous negative health equity implications of petroleum extraction, mining and large-scale agroindustry – all aggressively promoted by the Correa government as sources of funds to finance PNBV – suggest a need to look beyond traditional socially-oriented sectors such as health and education in attempting to achieve health in *all* policies [37]. They also bring into focus a range of possible contexts that could have strengthened the health equity approach in Ecuador. For example, the federal government could have used a broader intersectoral approach that integrates regional and local levels of government to include more stakeholders – both policy sectors and voices from the community – to address more local equity issues. The federal government also could have included specific objectives related to health inequities to strengthen the likelihood that action on social determinants of health would have benefited the well-being of marginalized populations. More fundamentally, the model of development that underlay PNBV could have been less reliant on financing from taxes on industries that have a conflict of interest with health equity [37].

In terms of limitations of this study, although we did include key informants from outside of the government (including academics and activists/advocates) and several pieces of literature authored by individuals external to government, some informants who were government employees may have been more likely to provide information about the implementation of HiAP that was favourable of the Correa administration, who remained in power at the time of our interviews (see Riofrancos [38] on ways in which government employees in the Correa years reproduced but sometimes resisted ‘extractivist’ policy orientations). This may have contributed to fewer thick mechanisms being identified in our data about the limitations of PNBV as a health equity intervention discussed above. Although we have tried to integrate some of the recent literature to provide a fuller understanding of the context of PNBV in Ecuador, future research could more directly examine mechanisms related to those factors of government.

A key strength of this study is the inclusion of interviews with diverse stakeholders, including politicians and civil servants from different government sectors as well as academics and activists/advocates. By analysing interviews and literature specific to Ecuador, we refined the conceptual framework described in Shankardass et al. [9] by identifying context specific mechanisms that supported or hindered HiAP implementation. We also used systems theory to analyse the system components that influence the sustainable implementation of HiAP. Future research is needed to uncover mechanisms that support or hinder HiAP implementation in countries with different political traditions or ideologies. Future studies should also look at the impact of HiAP for marginalized groups within the implicated jurisdiction’s borders to ensure that ‘equity’ is achieved.

## Conclusions

In conclusion, our realist case study approach uncovered mechanisms specific to the influence of political elites, opposition, motivation, and funding on the outcome of buy-in for HiAP implementation. Our systems framework highlighted how the executive subsystem and, in particular, political elites in Ecuador were deeply influential in achieving buy-in. Overall, this study contributes to the HiAP and policy literature by explaining how, why, and under what circumstances HiAP was implemented in Ecuador.

## Data Availability

The datasets generated and/or the summary tables from our analysis during the current study are not publicly available due to the sensitivity of confidential interviews with government employees, but they are available from the corresponding author in a de-identified manner upon reasonable request. The semi-structured interview guide is also available from the corresponding author.

## Abbreviations

BV: Buen vivir
Covid-19: Coronavirus disease/Severe acute respiratory syndrome coronavirus 2
CMO: Context-mechanism-outcome
IMF: International Monetary Fund
ISA: Intersectoral action
PNBV: Plan Nacional para el Buen Vivir
HiAP: Health in All Policies
SENPLADES: Secretaría Nacional de Planificación y Desarrollo
